# Publisher Correction: Long-term measles antibody profiles following different vaccine schedules in China, a longitudinal study

**DOI:** 10.1038/s41467-023-38167-4

**Published:** 2023-04-28

**Authors:** Qianli Wang, Wei Wang, Amy K. Winter, Zhifei Zhan, Marco Ajelli, Filippo Trentini, Lili Wang, Fangcai Li, Juan Yang, Xingyu Xiang, Qiaohong Liao, Jiaxin Zhou, Jinxin Guo, Xuemei Yan, Nuolan Liu, C. Jessica E. Metcalf, Bryan T. Grenfell, Hongjie Yu

**Affiliations:** 1 Shanghai Institute of Infectious Disease and Biosecurity, Fudan University, Shanghai, China; 2 School of Public Health, Fudan University, Key Laboratory of Public Health Safety, Ministry of Education, Shanghai, China; 3 Department of Epidemiology and Biostatistics, University of Georgia, Athens, GA USA; 4 Hunan Provincial Center for Disease Control and Prevention, Changsha, China; 5 Laboratory for Computational Epidemiology and Public Health, Department of Epidemiology and Biostatistics, Indiana University School of Public Health, Bloomington, IN USA; 6 Dondena Centre for Research on Social Dynamics and Public Policy, Bocconi University, Milan, Italy; 7 Department of Ecology and Evolutionary Biology, Princeton University, Princeton, NJ USA; 8 Princeton School of Public and International Affairs, Princeton University, Princeton, NJ USA

**Keywords:** Viral infection, Epidemiology, Vaccines, Measles virus

Correction to: *Nature Communications* 10.1038/s41467-023-37407-x, published online 29 March 2023

The original version of this article contained an error in the legend of Figure 1. The text “In addition, we revised the baseline age and the follow-up rates for participants in the child cohort due to the decimal point retention in (**B**).” was erroneously included. This text has been removed in both the PDF and HTML versions of the Article.

