# Supplementary material for: Long-term measles antibody profiles following different vaccine schedules in China, a longitudinal study
Source: Nat Commun. 2023 Mar 29;14:1746. doi: 10.1038/s41467-023-37407-x (PMC10054217; doi:10.1038/s41467-023-37407-x)
Supplement: Supplementary file 1 — Supplementary Information [file 41467_2023_37407_MOESM1_ESM.pdf]

# Long-term measles antibody profiles following different vaccine schedules in China, a longitudinal study

## Appendix

Qianli Wang[1,9], Wei Wang[2,9], Amy K Winter[3,9], Zhifei Zhan[4,9], Marco Ajelli[5], Filippo Trentini[6], Lili Wang[2], Fangcai Li[4], Juan Yang[2], Xingyu Xiang[4], Qiaohong Liao[2], Jiaxin Zhou[2], Jinxin Guo[2], Xuemei Yan[2], Nuolan Liu[2], C Jessica E Metcalf[7,8], Bryan T Grenfell[7,8], Hongjie Yu[1,2]

**Author Affiliations:**

1. Shanghai Institute of Infectious Disease and Biosecurity, Fudan University, Shanghai, China
2. School of Public Health, Fudan University, Key Laboratory of Public Health Safety, Ministry of Education, Shanghai, China
3. Department of Epidemiology and Biostatistics, University of Georgia, Athens, GA, USA
4. Hunan Provincial Center for Disease Control and Prevention, Changsha, China
5. Laboratory for Computational Epidemiology and Public Health, Department of Epidemiology and Biostatistics, Indiana University School of Public Health, Bloomington, IN, USA
6. Dondena Centre for Research on Social Dynamics and Public Policy, Bocconi University, Milan, Italy
7. Department of Ecology and Evolutionary Biology, Princeton University, Princeton, NJ, USA
8. Princeton School of Public and International Affairs, Princeton University, Princeton, NJ, USA
9. These authors contributed equally: Qianli Wang, Wei Wang, Amy K Winter, Zhifei Zhan.

Correspondence to: Prof. Hongjie Yu, Shanghai Institute of Infectious Disease and Biosecurity, Fudan University, Shanghai, 200032, China. Email: yhj@fudan.edu.cn

## Table of contents

Supplementary Note 1. A brief introduction to the two community-based longitudinal cohorts and the procedure of collecting information for cohort participants ........ 4
Supplementary Note 2. The procedures of ELISA assay ........ 5
Supplementary Note 3. Estimating the family-level socioeconomic status index ........ 5
Supplementary Note 4. Adjusting for the capability of ELISA in detecting near-threshold antibody concentrations ........ 6
Supplementary Note 5. Quantifying the impact of potential drivers on the measles-specific IgG antibody concentration ........ 7
Supplementary Note 6. Fitting measles-specific IgG antibody concentration 8
Supplementary Table S1. Characteristics comparison between children whose immunization records were available and those whose records were not. ........ 9
Supplementary Table S2. Factors associated with measles-specific IgG antibodies using a generalized linear mixed model. ........ 10
Supplementary Table S3. Random effects and goodness-of-fit in the generalized linear mixed model. ........ 11
Supplementary Table S4. Goodness-of-fit statistics for generalized additive mixed model while determining the kinetics of measles-specific IgG antibodies prior to and following a two-dose schedule of measles-containing vaccine at 8 and 18 months of age. ........ 12
Supplementary Figure S1. Measles epidemics in Anhua County, Hunan Province, during 2003-2018. ........ 13
Supplementary Figure S2. Timing of immunization among participants with complete immunization records (N=1,431). ........ 14
Supplementary Figure S3. Time at blood sampling and observed measles antibody concentrations by the availability of immunization records. ........ 15
Supplementary Figure S4. Observed individual antibody trajectories in children receiving a single-dose or two-dose schedule of measles-containing

vaccine that followed the recommendation of China's national immunization program. .......... 16
Supplementary Figure S5. Fitted profiles for measles antibody concentrations using different protective thresholds. .......... 17
Supplementary Figure S6. Measles-specific antibody dynamics following the first dose of measles-containing vaccine at 8 months by use of repeatedly drawn bootstrap samples. .......... 18
Supplementary Figure S7. Measles-specific antibody dynamics following a two-dose schedule of measles-containing vaccine at 8 and 18 months of age using different statistical models. .......... 19
Supplementary Figure S8. Simulated evolution of IgG antibody concentration following a single-dose or two-dose schedule of measles-containing vaccine that followed the recommendation of China's national immunization program. .......... 20
Supplementary Figure S9. Observed measles antibody concentrations by vaccination schedule. .......... 21
Supplementary Figure S10. The estimated cumulative incidence of seroreversion among children receiving the first dose of MCV at 8 months of age and subsequent one or two doses between the ages of 8 months and 5 years, adjusting for the capability of ELISA in detecting near-threshold antibody concentrations. .......... 22
Supplementary Figure S11. Cumulative incidence of seroreversion among children receiving the first dose of MCV at 8 months of age and subsequent one or two doses between the ages of 8 months and 5 years, using a protective threshold of 300 mIU/ml. .......... 23
References .......... 24

## Supplementary Note 1. A brief introduction to the two community-based longitudinal cohorts and the procedure of collecting information for cohort participants

*Cohort of mother-neonate pairs*

A total of 1,066 pairs of neonates and mothers (from 1,054 mothers) were enrolled by well-trained nurses in the six local hospitals in Anhua County, Hunan Province, China. Neonates who were born after September 20, 2013, and their mothers had resided in the study sites for 3 months or longer were eligible for enrolment. Umbilical cord blood samples (2 ml) were collected at birth (baseline, September 2013-October 2015) from neonates, in addition to venous blood samples at months 2, 4, 6, 12, 24, and 36. A detailed description of the paired mother-neonate cohort is reported elsewhere (1).

*Cohort of children aged 1-9 years*

A total of 4,188 children aged 1-9 years were randomly enrolled by well-trained project personnel and/or village doctors in the three townships (Tianzhuang, Jiangnan, and Qingtang) in Anhua County, Hunan Province, China. Children were eligible for inclusion if they were 1-9 years of age at enrolment and resided in the study sites in the last ≥3 months. A total of six follow-up visits (i.e., follow-up visit 1: February-March 2014; follow-up visit 2: August-October 2014; follow-up visit 3: March 2015; follow-up visit 4: August-October 2015; follow-up visit 5: March 2016; follow-up visit 6: August-November 2016) were conducted for enrolled children in accordance with the epidemic seasons of hand, foot, and mouth disease (HFMD) from February-March 2014 to August-November 2016. A venous blood sample (2 ml) was drawn from the participant at baseline and at six follow-up visits over three years. A detailed description of the cohort of children aged 1-9 years is reported elsewhere (2).

*Procedure of collecting baseline characteristics and vaccination cards*

In both cohorts, enrolled women or caregivers of children were interviewed face-to-face and completed a questionnaire at baseline to obtain the general characteristics of the children. Information regarding the vaccination history of

all recruited children was collected from vaccination cards, which serve as the official and legal documents required to enrol children in school and to show that their vaccination schedules are complete (3).

In the cohort of mother-neonate pairs, information on vaccination status recorded on vaccination cards was collected by trained investigators during the final three follow-up visits in September 2016, March 2018 and August 2018. In the cohort of children aged 1-9 years, the vaccination history documented on vaccination cards was collected by trained investigators or village doctors at the final follow-up visit. Two reviewers independently extracted data from the vaccination cards and assessed the data quality.

**Supplementary Note 2. The procedures of ELISA assay**

In accordance with the manufacturer's instructions, all samples were diluted 1:100 in assay diluents before testing. The negative control and standard sera (from the kit) were ready to use without further dilution. The resulting optical density values were converted into concentration units (mIU/mL) based on a calibration curve generated from standard serum using SERION software.

**Supplementary Note 3. Estimating the family-level socioeconomic status index**

The family-level socioeconomic status (SES) index is defined as the sum of the z score of education level, employment status, occupational status and annual household income, which was calculated as z scores for each variable and then summed into an overall index. The four variables used to estimate the family-level SES are shown in Supplementary Table 1. The range of the SES index was -3.40 to 5.12 (n=346), with a mean of 0.05 and standard deviation (SD) of 1.56. The index was then divided into tertiles, i.e., low SES=1st tertile [-0.40, -0.95), middle SES=[-0.95, 0.60), and high SES=[0.60, 5.12].

**Supplementary Note 4. Adjusting for the capability of ELISA in detecting near-threshold antibody concentrations**

To adjust for the capability of ELISA in detecting near-threshold antibody concentrations (defined here as values between a protective threshold ±50 mIU/ml) (4), we first assessed the overall consistency between antibody concentrations by ELISA and the "gold standard" plaque reduction neutralization test (PRNT). A subset of 120 serum samples including positive, equivocal, and negative IgG ELISA results were evaluated using the PRNT.

*Procedure of the plaque reduction neutralization test*

Sera were tested for measles neutralizing antibody using an adapted PRNT as previously described (5). In brief, sera were heated at 56°C for 30 minutes to inactivate the complement. Serial fourfold dilutions of serum samples and an in-house positive control serum from 1:4 to 1:4,096 were prepared, and each 120μL serum sample was mixed with an equal volume of measles virus (strain Edmonston, VR-24, American Type Culture Collection) containing 35 ± 5 plaque-forming units (PFUs) for 1.5 hours at 37°C, and 5% $CO_2$. The final dilution of the test sera ranged from 1:8 to 1:8192.

Following incubation, the serum-virus mixture (100μL/well) was transferred onto a confluent layer of Vero cells (CCL-81, American Type Culture Collection) in 24-well plates in duplicate. After incubating for 1 hour at 37°C, and 5% $CO_2$, the inoculum was removed, and each well was overlaid with a final concentration of 2% carboxymethylcellulose (low viscosity, Sigma-Aldrich, St. Louis, MO). At least six wells with non-neutralized challenge virus were included in every assay run as a virus control.

After being incubated for 4 days, the monolayers were stained in situ by adding 0.33% neutral red (Sigma-Aldrich, St. Louis, MO) in minimal essential medium (MEM) supplemented with 2% fetal bovine serum (FBS) (Thermo Scientific, Waltham, MA) to the overlay medium, and the plates were incubated for an additional day. Following the final incubation step, the stained overlay medium

was removed, and the monolayers were fixed with 10% formalin and air-dried in a biosafety cabinet. The number of plaques in each well was counted manually. The average PFU counts in duplicate wells were taken to determine the titer. The PRN titer was defined as the serum dilution that would reduce the number of plaques by 50% and the 50% endpoint was determined by the Karber formula. A PRN titer of ≥120 indicated measles seroprotection and is considered to be a level of antibody protective against measles disease. (6) All PRNT experiments were performed in an approved Biosafety Level 2 setting.

*Statistical analysis*

We found a significant correlation between serological results by the two assays ($r$=0.844, $p$<0.001), and obtained a concordance rate of 95.8% (115/120) between the two assays (differences in the number of susceptible individuals detected by ELISA and PRNT test: 18 vs. 23) (**Supplementary Figure 10A**), which is consistent with a previously published study (95.1%) (7). To account for this, we selected 4.2% (which was derived from 1-95.8%) of children with positive antibody concentrations near the threshold and directly changed their susceptibility status (i.e., susceptible or protective) from "protective" to "susceptible". Based on the updated data on children's susceptibility status, we reran the analysis of the cumulative incidence of seroreversion in IgG antibodies following different vaccination schedules.

## Supplementary Note 5. Quantifying the impact of potential drivers on the measles-specific IgG antibody concentration

Generalized linear mixed models (GLMMs) were used to explore factors associated with log-transformed measles-specific IgG antibody concentrations. Incorporating baseline characteristics, age and vaccination status (i.e., pre-vaccination, after a single dose vaccination at 8 months, and after double dose vaccination at 18 months) of each child participant produced the following model:

$$g(\mathrm{E}[y_i]) = \alpha + \beta_1 age_i + \beta_2 sex_i + \beta_3 \mathrm{gestational\_age}_i + \beta_4 \mathrm{mode\_delivery}_i + \beta_5 \mathrm{birth\_weight}_i + \beta_6 \mathrm{breastfeeding}_i + \beta_7 vax\_status_i + u_0$$

where $g$ is the log link function, and $y_i$ is the IgG antibody concentration for the $i$th individual. $\alpha$ is the intercept, and $age_i$ is the actual effect of age on the log of antibody concentration. $sex_i$, $\text{gestational\_age}_i$, $\text{mode\_delivery}_i$, $\text{birth\_weight}_i$ and $vax\_status_i$ refer to the sex, gestational age, mode of delivery, birth weight and vaccination status for the $i$th individual, whereas $\text{breastfeeding}_i$ indicates whether individual $i$ was breastfed before 6 months of age. $u_0$ denotes the random effects attributed to a child following repeated measurements.

**Supplementary Note 6. Fitting measles-specific IgG antibody concentration**

In light of the association between IgG antibodies and children's age and vaccination status, which was revealed by the above GLMM model, generalized additive mixed models (GAMMs) were used to fit log-transformed measles-specific IgG antibody concentrations (i.e., the kinetic model of antibody decay) across ages and by vaccination status. The model specification is:

$$g(\mathrm{E}[y_i]) = \alpha + \beta_1 s(age_i) + \beta_2 vax\_status_i + u_0$$

where $g$ is the log link function, and $y_i$ is the IgG antibody concentration for the $i$th individual. $\alpha$ is the intercept, and $s(age_i)$ is a smooth function used to characterize the actual effect of age on the log of individual-specific antibody concentration. $vax\_status_i$ refers to children's vaccination status, i.e., pre-vaccination, after a single dose vaccination at 8 months, and after double-dose vaccination at 18 months. $u_0$ denotes the random effects attributed to a child following repeated measurements. In sensitivity analyses, we also performed generalized additive models (GAMs) by removing random effects (i.e., $\mathrm{g}(\mathrm{E}[y]) = \alpha + \beta_1 s(age) + \beta_2 vax\_status$) so as to assess the goodness-of-fit of the GAMM models.

**Supplementary Table S1. Characteristics comparison between children whose immunization records were available and those whose records were not.**

| Characteristics | Total | Children without immunization records (n=888) | Children with immunization records (n=1,741) | p-value |
|---|---|---|---|---|
| Age at baseline, months or years | | | | |
| 0-7 months | 555 (21.1) | 137 (15.4) | 418 (24.0) | 3.0e-11 |
| 8-17 months | 121 (4.6) | 33 (3.7) | 88 (5.1) | |
| 18-24 months | 236 (9.0) | 73 (8.2) | 163 (9.4) | |
| 25 months-6 years | 1336 (50.8) | 465 (52.4) | 871 (50.0) | |
| 7-10 years | 381 (14.5) | 180 (20.3) | 201 (11.5) | |
| Township (n, %) | | | | |
| Qingtang (2.7 persons per 104 m$^2$) | 466 (17.7) | 124 (14.0) | 342 (19.6) | 9.7e-6 |
| Jiangnan (2.1 persons per 104 m$^2$) | 976 (37.1) | 310 (34.9) | 666 (38.3) | |
| Tianzhuang (1.5 persons per 104 m$^2$) | 1187 (45.2) | 454 (51.1) | 733 (42.1) | |
| Sex | | | | |
| Female | 1268 (48.2) | 408 (45.9) | 860 (49.4) | 0.102 |
| Male | 1361 (51.8) | 480 (54.1) | 881 (50.6) | |
| Gestational age, weeks | | | | |
| Preterm birth | 142 (5.4) | 49 (5.5) | 93 (5.3) | 0.333 |
| Full-term birth | 2451 (93.2) | 831 (93.6) | 1620 (93) | |
| Post-term birth | 36 (1.4) | 8 (0.9) | 28 (1.6) | |
| Mode of delivery (n, %) | | | | |
| Vaginal delivery | 1673 (63.6) | 571 (64.3) | 1102 (63.3) | 0.643 |
| Caesarean section | 956 (36.4) | 317 (35.7) | 639 (36.7) | |
| Birth weight, grams | | | | |
| Median, IQR | 3250 (3000-3500) | 3250 (3000-3500) | 3250 (3000-3500) | - |
| <2500 | 85 (3.2) | 25 (2.8) | 60 (3.4) | 0.106 |
| 2500 to <4000 | 2301 (87.5) | 767 (86.4) | 1534 (88.1) | |
| ≥4000 | 243 (9.2) | 96 (10.8) | 147 (8.4) | |
| Breast feeding | | | | |
| Yes | 2309 (87.8) | 782 (88.1) | 1527 (87.7) | 0.784 |
| No | 319 (12.1) | 105 (11.8) | 214 (12.3) | |
| Missing | 1 (0.1) | 1 (0.1) | - | |

Note that significance was tested using two-sided chi-square test or Fisher's Exact test.

**Supplementary Table S2. Factors associated with measles-specific IgG antibodies using a generalized linear mixed model.**

| Characteristics | No. of participants | Univariate analysis | | Multivariate analysis | |
|---|---|---|---|---|---|
| | | β (95%CI) | P-value | β (95%CI) | P-value |
| Age, months | 300 | 0.03 (0.03, 0.04) | 4.6e-30 | -0.03 (-0.04, -0.03) | 6.0e-18 |
| Sex | | | | | |
| Female | 156 | Reference | - | Reference | - |
| Male | 154 | -0.03 (-0.23, 0.17) | 0.777 | -0.02 (-0.20, 0.17) | 0.862 |
| Gestational age | | | | | |
| Preterm birth | 18 | Reference | - | Reference | - |
| Full-term birth | 272 | -0.09 (-0.51, 0.34) | 0.693 | 0.08 (-0.32, 0.48) | 0.682 |
| Post-term birth | 10 | -0.18 (-0.84, 0.48) | 0.596 | 0.03 (-0.59, 0.65) | 0.926 |
| Mode of delivery | | | | | |
| Vaginal delivery | 188 | Reference | - | Reference | - |
| Caesarean section | 112 | -0.03 (-0.24, 0.18) | 0.775 | -0.01 (-0.20, 0.18) | 0.927 |
| Birth weight, grams | | | | | |
| Log-transformed weight | 300 | -0.64 (-1.36, 0.09) | 0.087 | -0.45 (-1.14, 0.24) | 0.198 |
| Breast-feeding | | | | | |
| No | 30 | Reference | - | Reference | - |
| Yes | 270 | -0.08 (-0.43, 0.27) | 0.657 | -0.02 (-0.35, 0.30) | 0.892 |
| Number of doses* | | | | | |
| 0 (i.e., non-vaccination) | 204 | Reference | - | Reference | - |
| 1 (i.e., MCV1) | 203 | 1.97 (1.75, 2.19) | 1.2e-75 | 2.31 (2.08, 2.54) | 8.8e-94 |
| 2 (i.e., MCV2) | 279 | 1.93 (1.77, 2.09) | 5.6e-133 | 3.02 (2.71, 3.32) | 3.6e-108 |

*The number of participants may not total 300 due to repeated measurements for an individual.

Note that significance was tested using two-sided Wald test with α=0.05.

**Supplementary Table S3. Random effects and goodness-of-fit in the generalized linear mixed model.**

| | Value |
|---|---|
| Random effects for individual heterogeneity | |
| Variance | 0.244 |
| Standard deviation | 0.494 |
| $R^2$ | |
| Total | 0.404 |
| Fixed effects | 0.327 |
| Random effects | 0.077 |
| Adjusted $R^2$ | |
| Total | 0.400 |
| Fixed effects | 0.323 |
| Random effects | 0.078 |

**Supplementary Table S4. Goodness-of-fit statistics for generalized additive mixed model while determining the kinetics of measles-specific IgG antibodies prior to and following a two-dose schedule of measles-containing vaccine at 8 and 18 months of age.**

| Model* | $R^2$ | AIC (ΔAIC) | BIC | deviance | P-value |
|---|---|---|---|---|---|
| Generalized additive model | 0.631 | 4345.2 | 4382.3 | 1627.8 | - |
| Generalized additive mixed model | 0.634 | 4333.6 (-11.6) | 4375.9 | 1612.8 | 0.002 |

*Data from individuals who seroconverted after receiving the measles-containing vaccine were included in the two models.

Note that significance was tested using two-sided Wald chi-square test with $\alpha$=0.05.

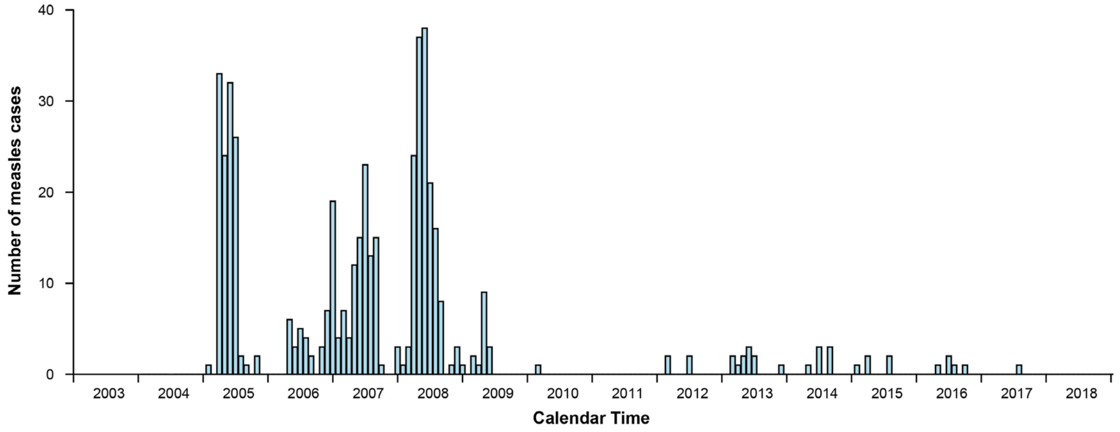


**Supplementary Figure S1. Measles epidemics in Anhua County, Hunan Province, during 2003-2018.**

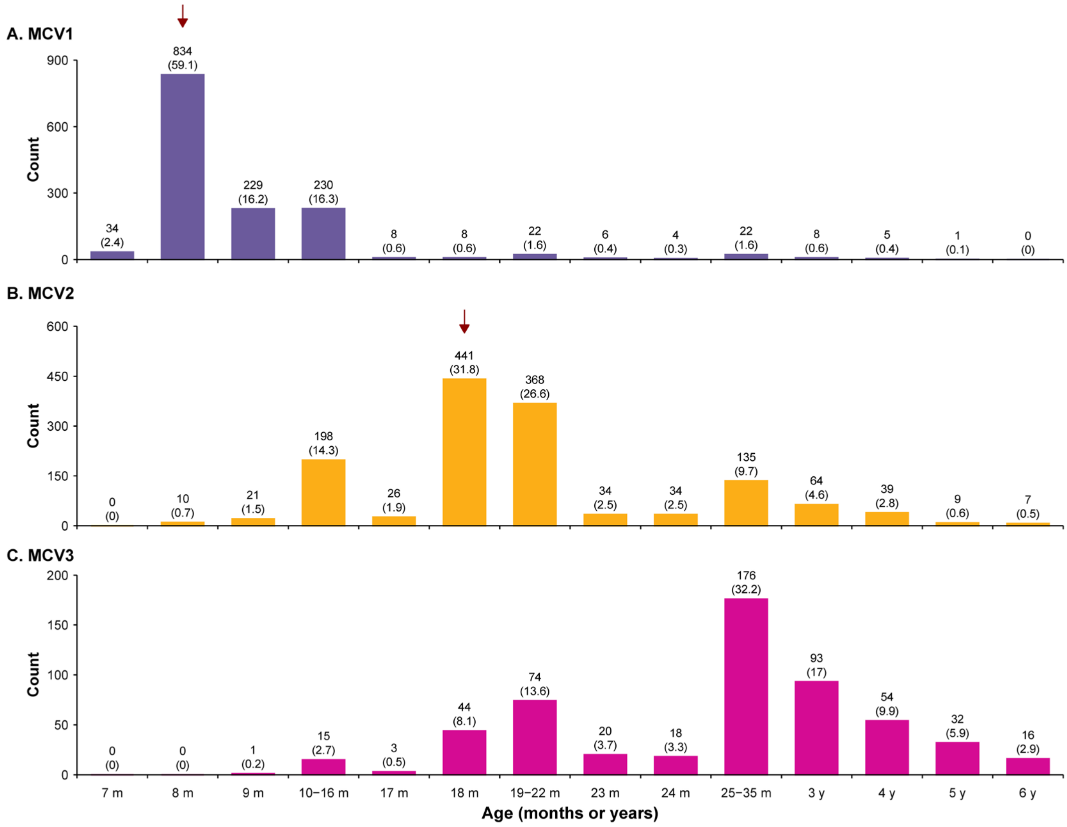


**Supplementary Figure S2. Timing of immunization among participants with complete immunization records (N=1,431).**

Note that the number above each bar refers to the number of individuals who received a given dose of measles-containing vaccine in a specific age group. Red arrows indicate the recommended age for the first and second doses of measles-containing vaccine in China.

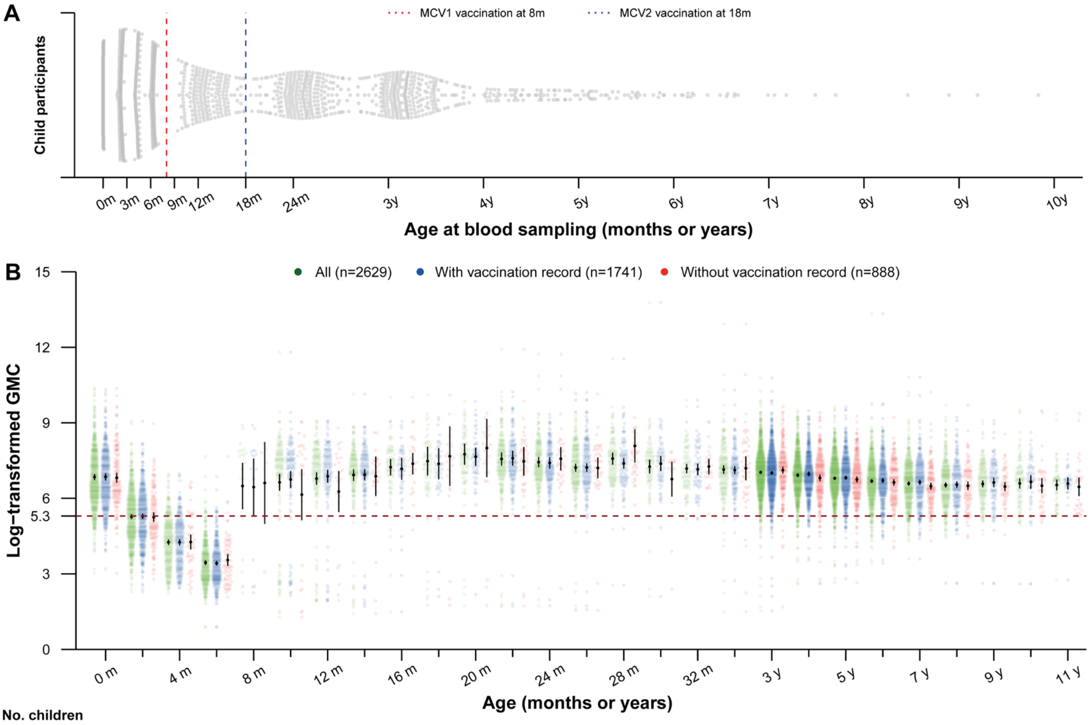


No. children

| | | | | | | | | | | | | | | | | | | | | | | | | | | | |
|---|---|---|---|---|---|---|---|---|---|---|---|---|---|---|---|---|---|---|---|---|---|---|---|---|---|---|---|
| Group 1 | 555 | 529 | 310 | 373 | 22 | 140 | 158 | 176 | 88 | 79 | 94 | 212 | 212 | 224 | 139 | 90 | 92 | 193 | 797 | 554 | 550 | 367 | 292 | 215 | 115 | 60 | 32 |
| Group 2 | 418 | 402 | 258 | 303 | 16 | 114 | 134 | 143 | 60 | 52 | 67 | 161 | 173 | 182 | 101 | 73 | 66 | 140 | 628 | 367 | 355 | 247 | 186 | 137 | 70 | 36 | 19 |
| Group 3 | 137 | 127 | 52 | 70 | 6 | 26 | 24 | 33 | 28 | 27 | 27 | 51 | 39 | 42 | 38 | 17 | 26 | 53 | 169 | 187 | 195 | 120 | 106 | 78 | 45 | 24 | 13 |

**Supplementary Figure S3. Time at blood sampling and observed measles antibody concentrations by the availability of immunization records.**

(A) The time of MCV administration and blood sampling for children with two routine MCV doses at 8 and 18 months; (B) Observed measles antibody concentrations by the availability of immunization records. Each grey point in Panel A refers to the time at blood sampling for each individual. In Panel B, black points with error bars refer to the log-transformed geometric mean concentrations and 95% confidence intervals; open circles refer to the observed log-transformed antibody concentration for each individual; green (Group 1), blue (Group 2) and red (Group 3) colours refer to all participants and children with and without immunization records, respectively. The numbers below the panels are the number of individuals involved in each group.

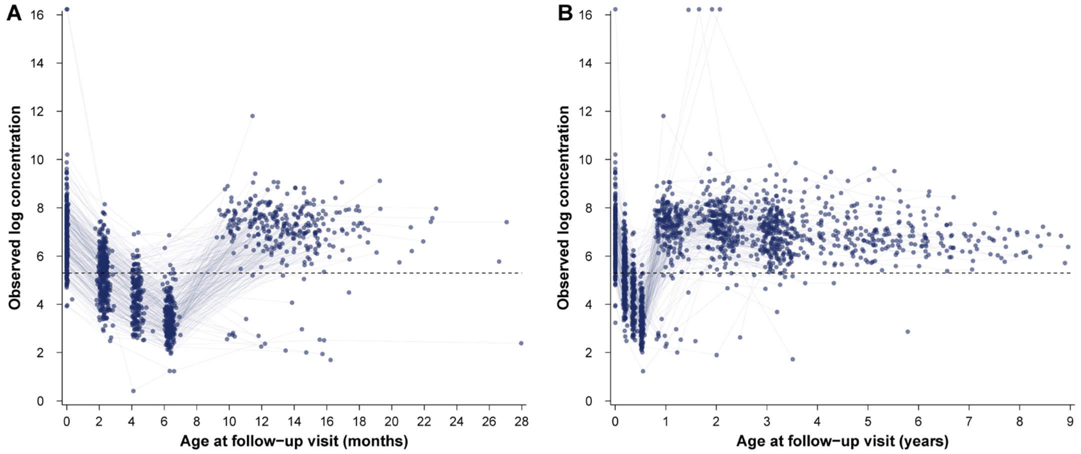


**Supplementary Figure S4. Observed individual antibody trajectories in children receiving a single-dose or two-dose schedule of measles-containing vaccine that followed the recommendation of China's national immunization program.**

(A) Children with a single-dose schedule at 8 months of age; (B) Children with a two-dose schedule at 8 and 18 months of age.

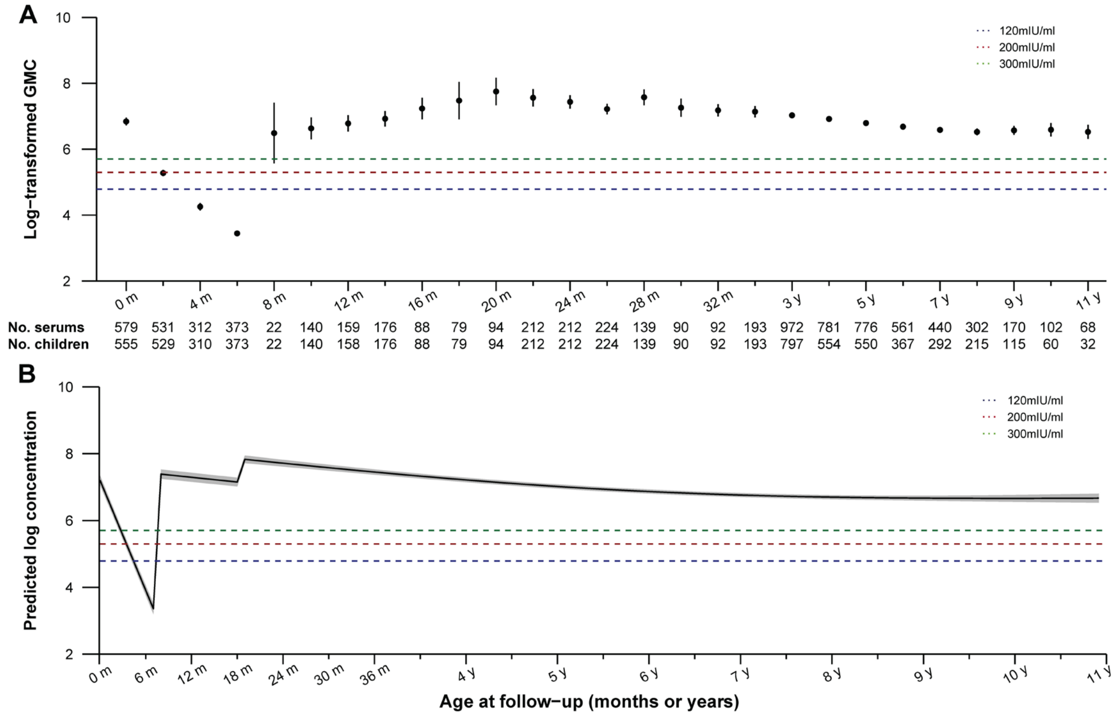


**Supplementary Figure S5. Fitted profiles for measles antibody concentrations using different protective thresholds.**

(A) Observed and (B) predicted log-transformed geometric mean concentrations among all participants (N=2,629). Points in Panels A refer to the log-transformed geometric mean concentrations. The thick curve in Panels B is the predicted mean value of log-transformed concentrations by use of generalized additive mixed model. Error bars and shaded areas show 95% confidence intervals.

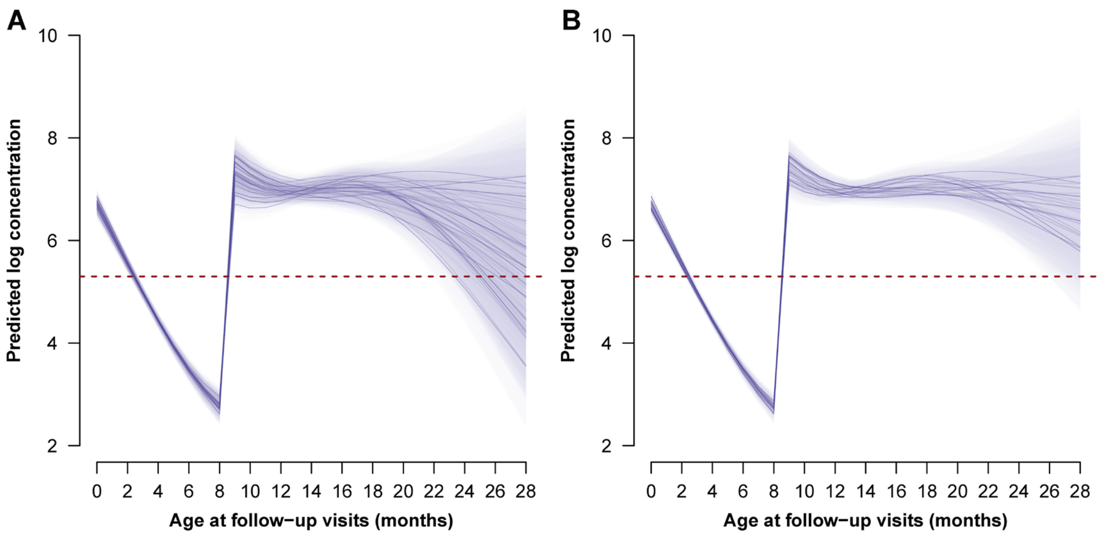


**Supplementary Figure S6. Measles-specific antibody dynamics following the first dose of measles-containing vaccine at 8 months by use of repeatedly drawn bootstrap samples.**

(A) Before and after (B) excluding data from a seronegative individual at 26-28 months who failed to seroconvert after MCV1 vaccination. Note that each line represents the fitted antibody concentration using a set of repeatedly drawn bootstrap samples, and shaded areas show 95% confidence intervals for each fitted antibody concentration.

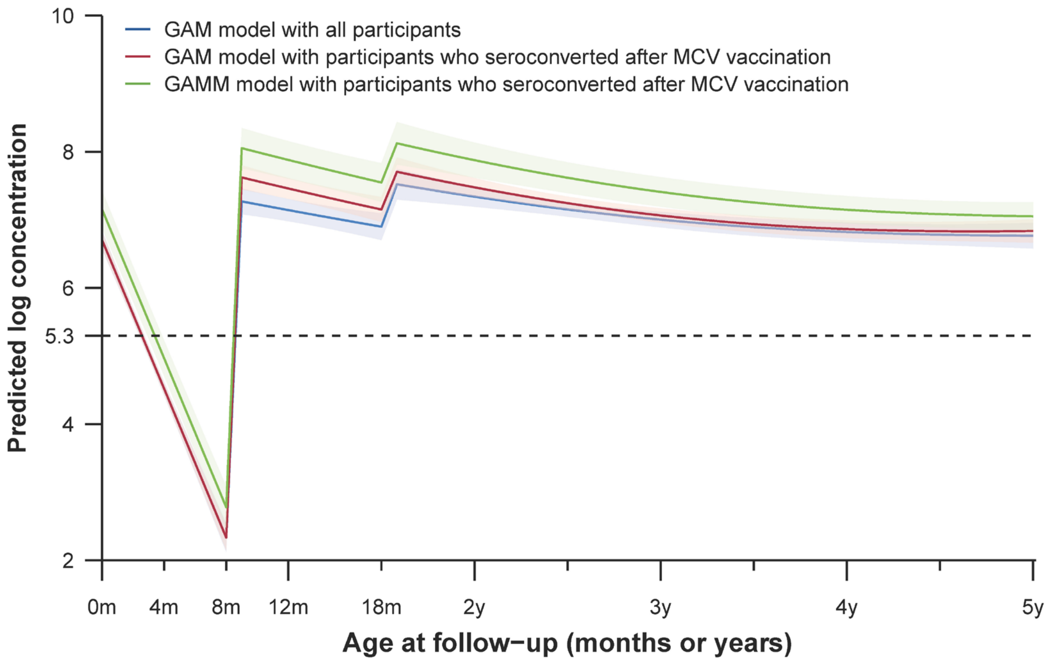


**Supplementary Figure S7. Measles-specific antibody dynamics following a two-dose schedule of measles-containing vaccine at 8 and 18 months of age using different statistical models.**

Note that the thick curves are the predicted mean value of log-transformed concentrations. Shaded areas show 95% confidence intervals.

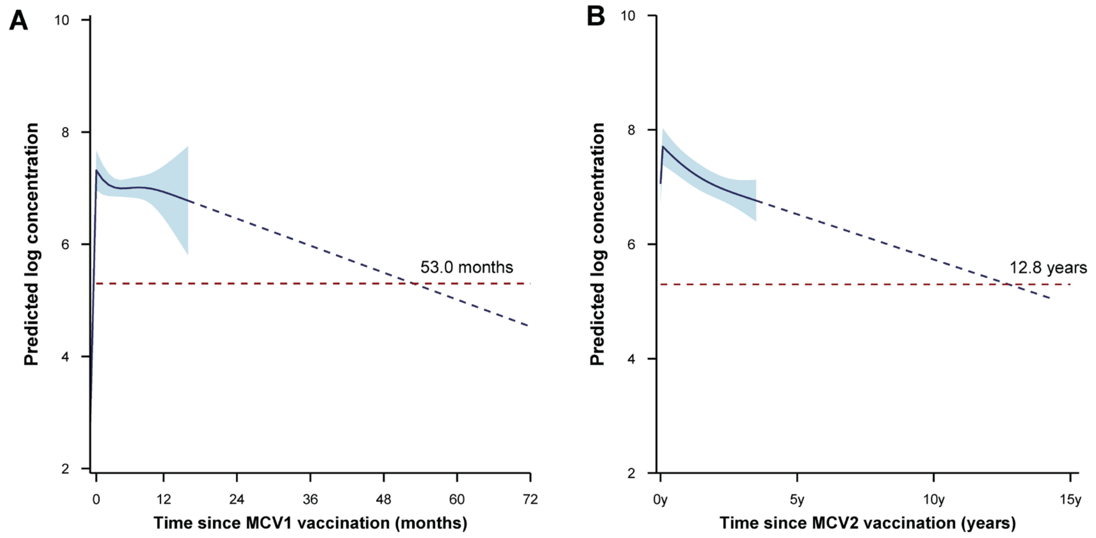


**Supplementary Figure S8. Simulated evolution of IgG antibody concentration following a single-dose or two-dose schedule of measles-containing vaccine that followed the recommendation of China's national immunization program.**

(A) Single-dose schedule at 8 months of age; (B) Two-dose schedule at 8 and 18 months of age. Note that blue and red dashed lines refer to the predicted log-transformed concentration by use of generalized additive mixed models and the protective thresholds of 200 mIU/ml, respectively.

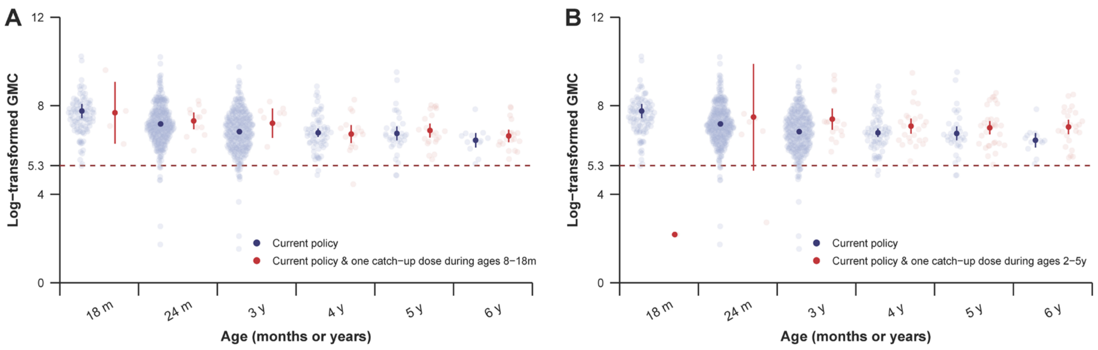


**A**

| No. children | | | | | | |
|---|---|---|---|---|---|---|
| Group 1 | 106 | 302 | 289 | 71 | 36 | 13 |
| Group 2 | 4 | 10 | 8 | 12 | 12 | 10 |

**B**

| No. children | | | | | | |
|---|---|---|---|---|---|---|
| Group 1 | 106 | 302 | 289 | 71 | 36 | 13 |
| Group 2 | 1 | 6 | 11 | 18 | 20 | 16 |

**Supplementary Figure S9. Observed measles antibody concentrations by vaccination schedule.**

(A) Log-transformed concentrations among children who received two routine MCV doses at 8 and 18 months of age (current policy) or received two routine MCV doses and a catch-up dose during ages 8-18 months. (B) Log-transformed concentrations among children who received two routine MCV doses or received two routine MCV doses and a catch-up dose during ages 2-5 years. Note that the protective threshold of 200 mIU/ml (i.e., the horizontal red line) was used in this analysis. Points in Panels A and B refer to the log-transformed geometric mean concentrations. Error bars show 95% confidence intervals. In the panel below, Group 1 represents children who received two routine MCV doses at 8 and 18 months (current policy); Group 2 represents children who received two routine MCV doses and a catch-up dose during ages 8 months and 5 years.

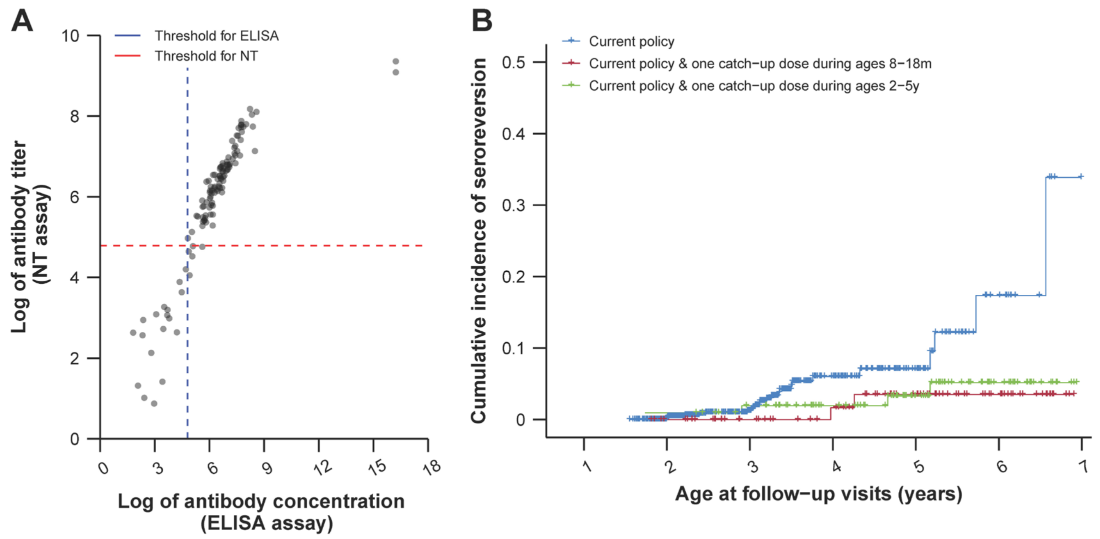


**Supplementary Figure S10. The estimated cumulative incidence of seroreversion among children receiving the first dose of MCV at 8 months of age and subsequent one or two doses between the ages of 8 months and 5 years, adjusting for the capability of ELISA in detecting near-threshold antibody concentrations.**

(A) Consistency between concentrations by neutralization test (NT) and ELISA; (B) The cumulative incidence of seroreversion among children receiving the first dose of MCV at 8 months of age and subsequent one or two doses between the ages of 8 months and 5 years, adjusting for the capability of ELISA in detecting near-threshold antibody concentrations. In Panel A, a threshold value of 120 was used to define seropositive individuals. In Panel B, blue, red and green lines refer to children who received two routine MCV doses at 8 and 18 months of age (i.e., current policy) or received two routine MCV doses and a catch-up dose between the ages of 8 months and 5 years.

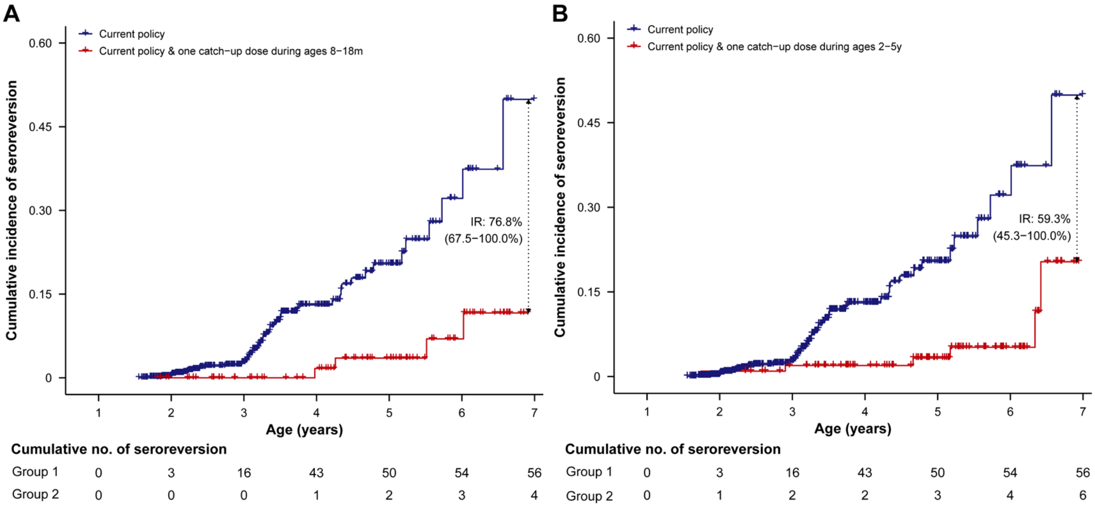


**Supplementary Figure S11. Cumulative incidence of seroreversion among children receiving the first dose of MCV at 8 months of age and subsequent one or two doses between the ages of 8 months and 5 years, using a protective threshold of 300 mIU/ml.**

(A) Cumulative incidence of seroreversion among children who received two routine MCV doses at 8 and 18 months of age (i.e., current policy) or received two routine MCV doses and a catch-up dose between 8 and 18 months of age. (B) Cumulative incidence of seroreversion among children who received two routine MCV doses at 8 and 18 months of age (i.e., current policy) or received two routine MCV doses and a catch-up dose between 2 and 5 years of age. In the bottom panel, Group 1 represents children who received two routine MCV doses at 8 and 18 months; Group 2 represents children who received two routine MCV doses and a catch-up dose between the ages of 8 months and 5 years. The abbreviation "IR" indicates the reduction in the cumulative incidence of seroreversion between the two groups.

## References


1. Wei X, Yang J, Gao L, Wang L, Liao Q, Qiu Q, et al. The transfer and decay of maternal antibodies against enterovirus A71, and dynamics of antibodies due to later natural infections in Chinese infants: a longitudinal, paired mother-neonate cohort study. The Lancet Infectious Diseases.
2. Yang J, Liao Q, Luo K, Liu F, Zhou Y, Zou G, et al. Seroepidemiology of enterovirus A71 infection in prospective cohort studies of children in southern China, 2013-2018. Nature Communications. 2022;13(1):7280.
3. He Y, Liu Y, Dai B, Zhao L, Lin J, Yang J, et al. Assessing vaccination coverage, timeliness, and its temporal variations among children in a rural area in China. Human vaccines & immunotherapeutics. 2021;17(2):592-600.
4. Ratnam S, Gadag V, West R, Burris J, Oates E, Stead F, et al. Comparison of commercial enzyme immunoassay kits with plaque reduction neutralization test for detection of measles virus antibody. J Clin Microbiol. 1995;33(4):811-5.
5. Cohen BJ, Audet S, Andrews N, Beeler J, test WHOwgomprn**.** Plaque reduction neutralization test for measles antibodies: Description of a standardised laboratory method for use in immunogenicity studies of aerosol vaccination. Vaccine. 2007;26(1):59-66.
6. Chen RT, Markowitz LE, Albrecht P, Stewart JA, Mofenson LM, Preblud SR, et al. Measles antibody: reevaluation of protective titers. J Infect Dis. 1990;162(5):1036-42.
7. Mao NY, Zhu Z, Jiang XH, Cui AL, Yan Z, Xu ST, et al. Comparison and Evaluation of Enzyme-linked Immunization Assay Kits with Plague Reduction Neutralization Test for Detection of Measles IgG Antibody. Chinese Journal of Vaccines and Immunization. 2009;15(3):215-8.
