# Supplementary material for: Long-term measles antibody profiles following different vaccine schedules in China, a longitudinal study
Source: Nat Commun. 2023 Mar 29;14:1746. doi: 10.1038/s41467-023-37407-x (PMC10054217; doi:10.1038/s41467-023-37407-x)
Supplement: Supplementary file 3 — Reporting Summary [file 41467_2023_37407_MOESM3_ESM.pdf]

nature portfolio

Corresponding author(s): Hongjie Yu
Last updated by author(s): Mar 1, 2023

Reporting Summary

Nature Portfolio wishes to improve the reproducibility of the work that we publish. This form provides structure for consistency and transparency in reporting. For further information on Nature Portfolio policies, see our Editorial Policies and the Editorial Policy Checklist.

Statistics

For all statistical analyses, confirm that the following items are present in the figure legend, table legend, main text, or Methods section.

n/a	Confirmed	
☐	☒	The exact sample size (*n*) for each experimental group/condition, given as a discrete number and unit of measurement
☐	☒	A statement on whether measurements were taken from distinct samples or whether the same sample was measured repeatedly
☐	☒	The statistical test(s) used AND whether they are one- or two-sided<br>*Only common tests should be described solely by name; describe more complex techniques in the Methods section.*
☐	☒	A description of all covariates tested
☐	☒	A description of any assumptions or corrections, such as tests of normality and adjustment for multiple comparisons
☐	☒	A full description of the statistical parameters including central tendency (e.g. means) or other basic estimates (e.g. regression coefficient) AND variation (e.g. standard deviation) or associated estimates of uncertainty (e.g. confidence intervals)
☐	☒	For null hypothesis testing, the test statistic (e.g. *F*, *t*, *r*) with confidence intervals, effect sizes, degrees of freedom and *P* value noted<br>*Give P values as exact values whenever suitable.*
☒	☐	For Bayesian analysis, information on the choice of priors and Markov chain Monte Carlo settings
☐	☒	For hierarchical and complex designs, identification of the appropriate level for tests and full reporting of outcomes
☒	☐	Estimates of effect sizes (e.g. Cohen's *d*, Pearson's *r*), indicating how they were calculated

Our web collection on statistics for biologists contains articles on many of the points above.

Software and code

Policy information about availability of computer code

Data collection	EXCEL was used to collected data.
Data analysis	All analyses were performed in R version 4.1.0. The R code to replicate the analyses is available on GitHub at https://github.com/Sueleaf/antibody-dynamics-against-measles.

For manuscripts utilizing custom algorithms or software that are central to the research but not yet described in published literature, software must be made available to editors and reviewers. We strongly encourage code deposition in a community repository (e.g. GitHub). See the Nature Portfolio guidelines for submitting code & software for further information.

Data

Policy information about availability of data

All manuscripts must include a data availability statement. This statement should provide the following information, where applicable:

- Accession codes, unique identifiers, or web links for publicly available datasets
- A description of any restrictions on data availability
- For clinical datasets or third party data, please ensure that the statement adheres to our policy

The data generated in this study have been deposited in the repository under https://github.com/Sueleaf/antibody-dynamics-against-measles (https://doi.org/10.5281/zenodo.7676630).

Human research participants

Policy information about studies involving human research participants and Sex and Gender in Research.

Reporting on sex and gender	All child participants were defined by sex. Relevant data are available within the paper and, in more detail, in the appendix file.
Population characteristics	We enrolled 2,629 children in this study, including 555 mother-neonate pairs and 2,074 children aged between 1 and 9 years. 555, 352, 354, 318, 335, 316, and 399 children were enrolled at birth and at 1, 2, 3, 4, 5, and 6-9 years of age, respectively. Of these, 1,268 (48.2%) children were female. The follow-up time ranged from 1 to 42 months (median 35.2 months, interquartile range (IQR) 29.3-36.2)
Recruitment	We used archived serum samples from two community-based longitudinal cohorts, including a cohort of mother-neonate pairs (n=1,066) and a cohort of children aged 1-9 years (n=4,188), that aimed to investigate the sero-epidemiological characteristics of paediatric enterovirus A71 infections in Hunan Province, China between September 2013 and September 2018.<br>A total of 1,066 pairs of neonates and mothers (from 1,054 mothers) were enrolled by well-trained nurses in the six local hospitals in Anhua County. We compared the characteristics of enrolled neonates with those from the same region who did not participate. Among neonates, sex and birthweight were similar.<br>A total of 4,188 children aged 1-9 years were randomly enrolled by well-trained project personnel and/or village doctors in the three townships in Anhua County. Children were eligible for inclusion if they were 1-9 years of age at enrolment and resided in the study sites in the last ≥3 months. Within each township, simple random sampling was used for the selection of children aged 1-9 years. The randomization procedure could have furthest reduced the risk of self-selection bias.
Ethics oversight	This study was approved by the Institutional review board from WHO Western Pacific Regional Office (2013.10.CHN.2.ESR), the Chinese Centre for Disease Control and Prevention (201224), and Fudan University (2019–05–0756), and written informed consent was obtained from all caregivers of participants.

Note that full information on the approval of the study protocol must also be provided in the manuscript.

Field-specific reporting

Please select the one below that is the best fit for your research. If you are not sure, read the appropriate sections before making your selection.

☒ Life sciences ☐ Behavioural & social sciences ☐ Ecological, evolutionary & environmental sciences

For a reference copy of the document with all sections, see nature.com/documents/nr-reporting-summary-flat.pdf

Life sciences study design

All studies must disclose on these points even when the disclosure is negative.

Sample size	Only a subsample of the original cohorts was included in this study. In particular, we tested 2,629 participants' serum samples for measles-specific IgG antibody; the included sera came from 555 mother-neonate pairs and 2,074 children aged between 1 and 9 years.
Data exclusions	Child participants were excluded from analysis only if they failed to receive 2 doses of measles vaccine at ages consistent with the recommendation of China's national immunization program.
Replication	Reported results were consistently in replicated Elisa assays with replicates generating similar results.<br>A subset of 120 serum specimens were performed in two independent assays: plaque reduction neutralization test (PRNT) and Elisa assay. We conducted the PRNT assay in biological duplicated wells in each dilution per samples. All replications were successful and gave similar results. Data were collected and checked by two of the authors. The statistical analyses were conducted and rechecked by two of the authors.
Randomization	2,629 child participants were randomly selected from original cohorts.
Blinding	Blinding during serum sample collection was not needed because conditions were well controlled.<br>There is only a unique number on the serum sample tube, and the identification information of the participant was blinded to experimental operator. Only anonymised data were used in the analysis.

Reporting for specific materials, systems and methods

We require information from authors about some types of materials, experimental systems and methods used in many studies. Here, indicate whether each material, system or method listed is relevant to your study. If you are not sure if a list item applies to your research, read the appropriate section before selecting a response.

Materials & experimental systems

n/a	Involved in the study	
☐	☒	Antibodies
☐	☒	Eukaryotic cell lines
☒	☐	Palaeontology and archaeology
☒	☐	Animals and other organisms
☒	☐	Clinical data
☒	☐	Dual use research of concern

Methods

n/a	Involved in the study	
☒	☐	ChIP-seq
☒	☐	Flow cytometry
☒	☐	MRI-based neuroimaging

Antibodies

Antibodies used

Quantitative results of measles-specific IgG antibody were obtained by using commercial ELISA kits (SERION ELISA classic measles virus IgG, Institut Virion/Serion GmbH, Wurzburg, Germany).

Validation

To evaluate the consistency between antibody concentrations measured through ELISA assay and the "gold standard" plaque reduction neutralization test (PRNT), a subset of 120 serum samples (including positive, equivocal, and negative IgG ELISA results) were re-evaluated using PRNT.

Eukaryotic cell lines

Policy information about cell lines and Sex and Gender in Research

Cell line source(s)

Cell line used in this study were obtained from ATCC (Vero, Cat. #CCL 81)

Authentication

The cell line used were not authenticated

Mycoplasma contamination

Cell line was not tested for mycoplasma contamination

Commonly misidentified lines
(See ICLAC register)

No commonly misidentified cell lines were used in the study
